# Effect of low sperm quality on progeny: a study on zebrafish as model species

**DOI:** 10.1038/s41598-019-47702-7

**Published:** 2019-08-01

**Authors:** Marta F. Riesco, David G. Valcarce, Juan Manuel Martínez-Vázquez, Vanesa Robles

**Affiliations:** 1 IEO, Spanish Institute of Oceanography, Planta de Cultivos el Bocal, Santander, 39012 Spain; 2 MODCELL GROUP, Department of Molecular Biology, Universidad de León, 24071 León, Spain

**Keywords:** Zebrafish, Spermatogenesis

## Abstract

Nowadays a decrease tendency in human sperm quality has been reported mainly in developed countries. Reproductive technologies have been very valuable in achieving successful pregnancies with low quality sperm samples. However, considering that spermatozoa molecular contribution is increasingly important in recent studies, it is crucial to study whether fertilization with low sperm quality could leave a molecular mark on progeny. This study explores the consequences that fertilization with low sperm quality may have on progeny, using zebrafish as a model. Good and bad breeders were established attending to sperm quality analyses and were individually tracked. Significant differences in fertilization and malformation rates were obtained in progenies between high and low quality sperm samples. Moreover an altered miR profile was found in the progenies of bad zebrafish breeders (upregulation of miR-141 and miR -122 in 24 hpf embryos) and as a consequence, some of their targets involved in male sex development such as *dmrt1*, suffered downregulation. Our results indicate that fertilizing with high sperm quality samples becomes relevant from a new perspective: to avoid molecular alterations in the progeny that could remain masked and therefore produce unexpected consequences in it.

## Introduction

It has traditionally been thought that reproductive and fertility outcomes mainly depend on the female factor^1,2^. The role of the male gamete has been limited to the narrow window of fertilization and the sperm cell considered almost like a simple vehicle for male DNA delivery to the embryo^3–5^. During recent years, several studies have demonstrated that this scenario is not real. The male contribution, far from being confined to DNA, has been extended to a wide variety of molecules including coding and non-coding RNAs^6–10^. MicroRNAs (miRNAs) are a class of small non-coding RNAs that inhibit post-transcriptional gene expression by binding to messenger RNA (mRNA) targets. Some miRNAs related to sperm quality and reproduction success have recently been described in different species and their high conservation grade among vertebrates highlighted^11–13^. Specifically in humans, the aberrant expression of miRNAs could be associated with different disorders including azoospermia, oligospermia and asthenozoospermia, among others (recently revised in Robles *et al*.^8^). However, in terms of quantity, the small contribution of spermatozoa when compared to the oocyte created skepticism regarding to the importance of these molecules of paternal origin beyond the particular moment of fertilization. Evidence of their importance has been provided in several studies that demonstrating that sperm cells can be a vehicle for certain phenotype transmission to the progeny^7,14–16^ pointing to molecules such as miRNAs as one of the possible mechanisms of transmission. Nowadays it is known that in oligo-asthenozoospermic human males (with low sperm count and sperm motility) there are several deregulated miRNAs when compared with normozoospermic males^17^. miR-122 is one of those that showed the highest fold changes in both asthenozoospermic and oligo-asthenozoospermic men together with miR-141 and miR-200a^18^. These types of studies demonstrate the high potential of miRNAs as future diagnostic tool for male infertility assessment. However, it could also be interesting to determine whether those low sperm quality samples that achieved fertilization could leave a permanent molecular mark on progeny, particularly taking into account that several studies point to a decrease in global sperm quality in human world population^19^.

A deep conservation of several site-specific miRNA editing events, including some from the common ancestor of mammals and bony fishes, has been reported^20^. Moreover, it has been demonstrated that zebrafish could be an excellent model for reproductive biology studies taking into account “the high similarities in reproductive functions and regulations between this small fish species and mammals”^21^. This species provides several advantages for research such as rapid development and high prolificacy, and several molecular and genetic tools are also available for it^21–23^.

Our hypothesis is that fertilization with low quality sperm samples could have other consequences rather than low fertility rates, leaving a mark on progeny either at molecular level or phenotypic level. In order to explore this possibility we use zebrafish as a model. Taking into account that the study of global and specific DNA methylation in crucial genes for spermatogenesis has recently achieved clinical relevance, a global and specific sperm methylation analysis has been performed in these experimental groups. DNA methylation deregulation is known to represent a possible explanation of increased incidence in syndromes related to genomic imprinting in Assisted Reproductive Technologies^24^.

In addition, sperm quality related miRs have also been analyzed in good and bad zebrafish breeders. All of these miRs (miR-122-5p, miR 141-3p and miR-200a-5p) were previously defined as molecular markers in human sperm^11^, and they are differentially present in normozoospermic when compared to asthenozoospermic and oligo-asthenozoospermic men^25^. Interestingly, miR-141-3p and miR-200a-5p were also proposed as sperm motility markers in zebrafish^26^ and other teleost species^13^. This study provides new evidences on the effects of fertilizing with low sperm quality samples on progeny.

## Methods

### Ethical statement

All experiments involving zebrafish were approved by the Spanish and institutional bioethical guidelines of the Animal Welfare Service, Government of Cantabria, and European Union Directive 2010/63/EU for the protection of animals for experimental uses. The authorization number for experimental procedures in this study is PI-10-16. All methods were performed in accordance with the relevant guidelines and regulations. Moreover, all those involved in the experiments have a FELASA class C permit for animal experimentation.

### Zebrafish maintenance

Adult zebrafish (*Danio rerio*) AB wild type were kept in 10 liter tanks under standard conditions in a circulating system (AquaticHabitats, Apopka, FL, USA) that continuously filters and aerates the system water to maintain the water quality required for a healthy aquatic environment. The fish were kept on a 14:10-h light: dark cycle and fed twice daily with dry commercial food (Aquatic Habitats, Apoka, FL, USA).

### Zebrafish tagging

The visible implant elastomer (VIE) tagging system was used to differentiate each zebrafish individual. A manual injection kit (60 mL) for VIE tags was purchased from Northwest Marine Technology (EEUU). Elastomer was prepared and visualized according to the manufacturer’s instructions^27^. Different tag colors and tag injection sites were employed to improve individual identification allowing individual tracking for male classification as good or bad breeders.

### Sperm collection by stripping

Sperm was collected from zebrafish males after immersion in 168 mg/mL tricaine methanosulfonate solution (MS-222) dissolved in system water. The urogenital pore was dried and sperm was collected with a pipette by bilateral abdominal pressure using fine forceps. The sperm was collected taking special care to avoid urine contamination. Prior to the analysis, the sperm was diluted 10-fold in a non-activating medium: Hank’s balanced salt solution (HBSS) at an osmolality of 300 mosmol/kg. Zebrafish males were transferred to fresh water tanks to recovery immediately after stripping. The samples were then stored at room temperature until motility analysis was performed.

### Fresh sperm quality assays for zebrafish male classification

**Figure 1:**
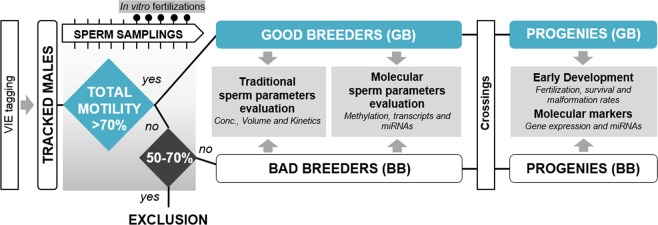
Experimental design. Schematic representation of the experimental group determination (good and bad zebrafish breeders) analyses carried out and parameters analyzed in each type of sample.

**Figure 2:**
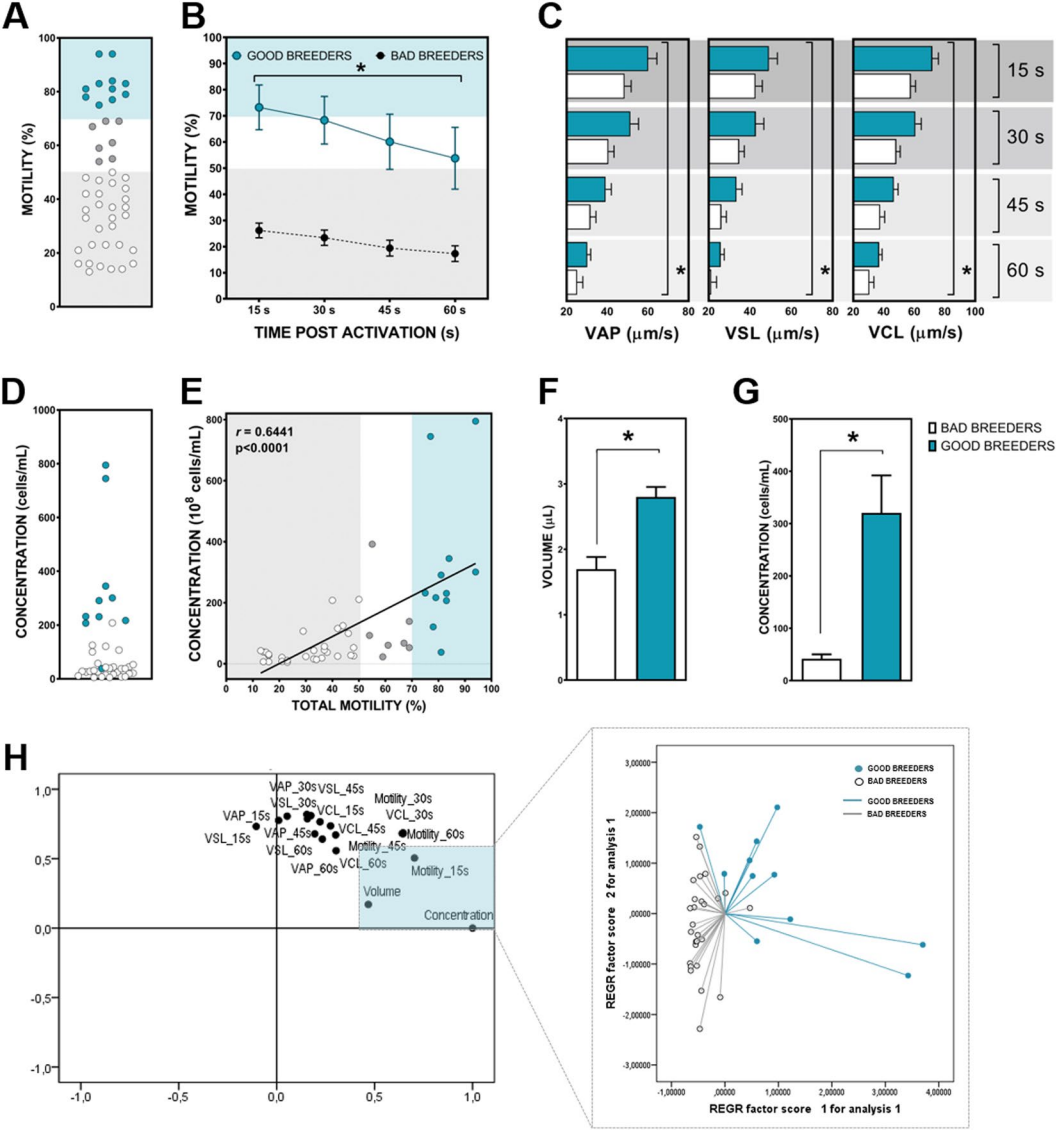
Sperm quality analyses in good and bad zebrafish breeders. (**A**) Representation of total population distribution in terms of total motility (%). (**B**) Total motility (%) at different post-activation times (15 s, 30 s, 45s and 60 s) in the experimental groups (good and bad breeders). (**C**) Kinetic parameters (µm/s) at different post-activation times (15 s, 30 s, 45s and 60 s) in good and bad breeders. (**D**) Representation of total population distribution in terms of concentration (cell/ml). (**E**) Correlation between total motility (%) and concentration (cell/ml) in the two experimental groups analyzed. (**F**) Volume (µl) of good and bad zebrafish breeders. (**G**) Concentration (cell/ml) of the two studied groups. (**H**) Principal Component Analysis including all sperm quality parameters in good and bad zebrafish breeders. Representation of our experimental groups in a principal component plane. Data are the mean ± SEM. Good zebrafish breeders are represented in blue and bad breeders in white. Asterisks represent significant differences (p < 0.05) between experimental groups. Analyses were performed in sperm samples from 48 individual males.

In this study, good and bad zebrafish breeders were established in terms of total motility according to previous studies that claimed that fertility was directly correlated with sperm motility in zebrafish^28,29^ (Fig. 1). Total sperm motility has been considered the most important parameter in sperm quality definition due to its high correlation with fertilization success in different fish species^28–30^. Sperm volume was measured using a 10 µl glass microcapillary. Motility and concentration analyses were performed activating 1 μl of sperm with 9 μl of system water (28 °C). Total motility was analyzed using computer assisted sperm analysis (CASA) and ISAS software (ISAS, Proiser R + D, S.L., Spain). Motility was assessed in a Makler chamber using a phase-contrast microscope (Nikon Eclipse Ts2R, Japan) with a 10x negative contrast objective and a digital camera set for 50 fps. The settings for CASA software were adapted for fish species. The following CASA parameters were analyzed: total motility, curvilinear velocity (VCL), straight line velocity (VSL) and average path velocity (VAP). Motility parameters were registered at 15 s, 30 s, 45 s and 60 s after sperm activation with water system. At least 200 spermatozoa were analyzed for each sample (3 fields per sample). If samples reported very low concentrations, more than three fields were captured. Forty-eight individual males were analyzed for experimental group set up (Fig. 2A). These sperm quality analyses were repeated 10 times for zebrafish male classification attending to total motility parameter (Fig. 1). After these analyses, we obtained a total of 11 good zebrafish males, 7 intermediate males, which were excluded, and 30 bad zebrafish males in terms of motility (Fig. 2A). In this sense, zebrafish tagging allowed the individual zebrafish tracking for zebrafish male classification.

### *In vitro* fertilization assays

Once the breeders were characterized according to sperm quality parameters, fertilization trials were done and fertility rates recorded together with malformation rates and survival during development. At molecular level, the relative expression of miRNAs altered in low sperm quality samples was analyzed in different developmental stages in the progeny. Moreover, some mRNA targets of such miRNAs were predicted and their relative expression measured in the embryos. For this purpose, tagged and classified zebrafish males in good and bad breeders were isolated from females in breeding tanks and food was withheld at the end of the afternoon before gamete collection. *In vitro* fertilization was performed according the protocol published on the ZIRC web site (https://zebrafish.org/documents/protocols.php). *In vitro* fertilization was carried out using the same pool of oocytes to avoid the bias that would produce the fertilization with female gametes of different quality. This way we can guarantee that any difference observed is consequence of sperm contribution rather than oocyte. To achieve this objective, an initial pool of eggs (around 200 eggs) was split into two batches to be fertilized with sperm from good and bad breeders using the same egg pool. Five different *in vitro* fertilization experiments (with a minimum of 3 females per pool) were carried out in each experimental group (Fig. 1).

### Molecular assays

#### Global and specific methylation

**Figure 3:**
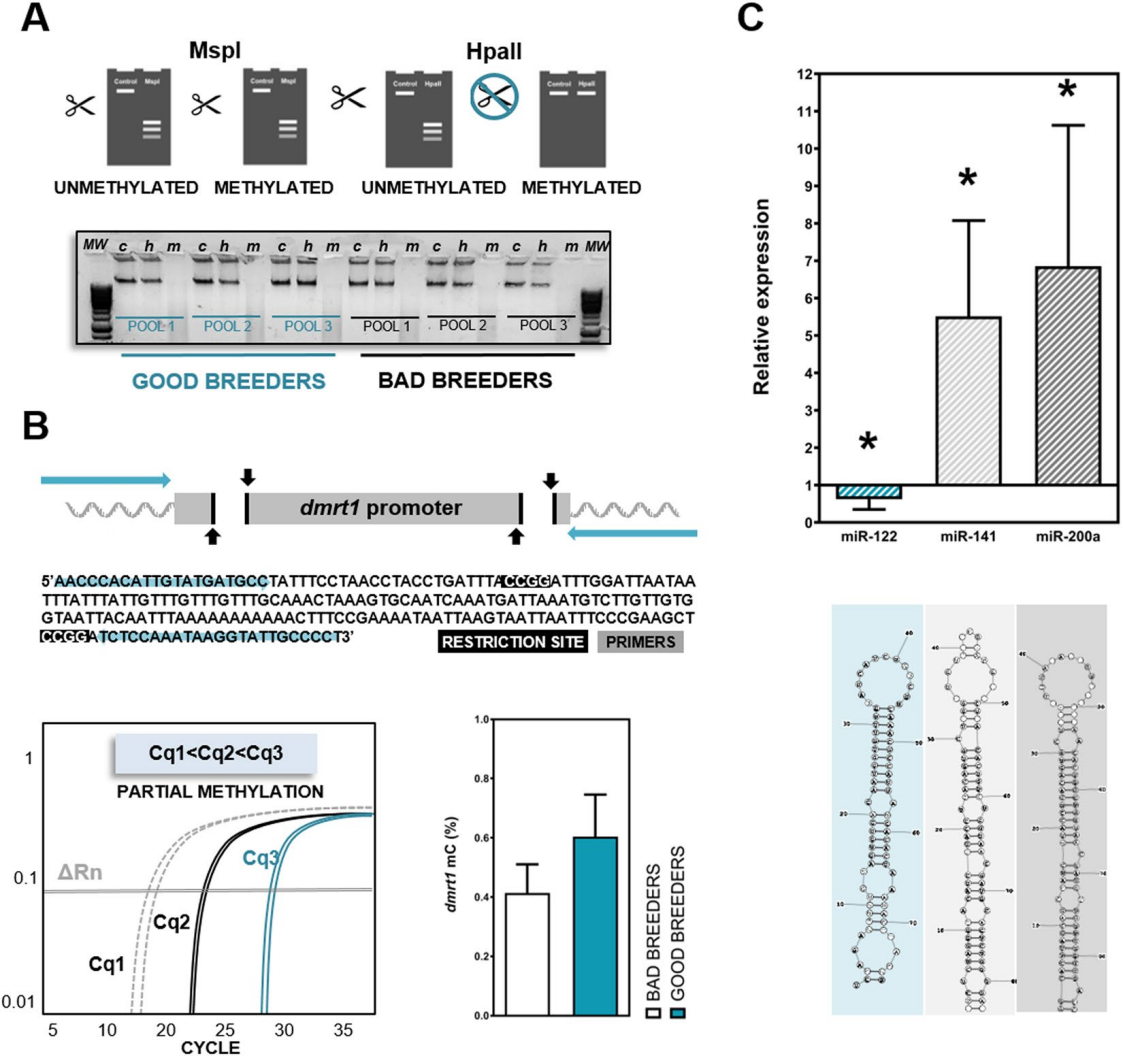
Sperm molecular analyses in good and bad zebrafish breeders. (**A**) Global methylation status of sperm DNA. Unmethylated plasmid DNA (kit control) is digested by both enzymes, whereas methylated plasmid DNA (kit control) is susceptible only to Epi MspI digestion. All the represented samples from good and bad breeders derived from the same experiment and gel. The full length-gel image with all samples and kit controls is available in Supplementary Fig. 1. (**B**) Specific methylation analysis in *dmrt1* promoter of good and bad zebrafish breeders. Schematic representation of zebrafish *dmrt1* promoter, sequence, primer design, restriction sites, pattern of methylation and percentage of methylcytosine obtained in each experimental group. (**C**) Structure and expression of each microRNA normalized against that of miR-92-3p was calculated for all samples using the 2^−ΔΔCt^ method. The figure shows expression of each microRNA in the bad breeders relative to that in good breeders, which was set to 1. Data are expressed as the mean ± SEM of 2^−ΔΔCt^ values from three independent experiments of three different pools of three males per group were analyzed. Asterisks represent significant differences (p < 0.05) between experimental groups.

DNA from different sperm samples was isolated using the GeneJET Genomic DNA Purification Kit according to the manufacture’s protocol. Sperm global methylation status was evaluated in both experimental groups (good and bad zebrafish breeders) using the EpiJET DNA Methylation Analysis Kit (MspI/HpaII). A simple digestion with two restriction enzymes (MspI and Hpa II) was performed for each experimental group for 1 h at 37 °C. Different control samples were included in the experiment (undigested DNA sample as negative control and completely methylated and unmethylated DNA). The reaction products were analyzed by DNA electrophoresis on a 1% agarose gel after digestion. Three experiments of three different pools of males (3 males/pool) were analyzed. Moreover, a locus specific method providing percent methylation information was performed. For this purpose, a CCGG particular locus (*dmrt1*) was analyzed by qPCR after restriction enzyme digestion. Firstly, a set primers that flank the CCGG site of interest was designed. One or two μL of product digestion (MspI and Hpa II) were employed to carry out a qPCR follow the manufacturer’s recommendations for qPCR reaction set-up and cycling conditions. 5-mC percentage was calculated using the formula below:

$$ \% \,{of}\,5 \mbox{-} {mC}=\frac{100}{{(1+E)}^{({Cq}2-{Cq}1)}}$$

where: Cq1 is the threshold cycle of “Undigested DNA” sample, Cq2 is the threshold cycle of “Digested with Epi HpaII” sample and Cq3 is the threshold cycle of “Digested with Epi MspI” sample and E is the PCR efficiency value (%) (Fig. 3B).

### miR and transcript quantification

#### miR and RNA isolation

The study of miRs was carried out in sperm and embryo samples (at 2–4 cell, blastula and 24 hours post fertilization stages) and mRNA analysis was performed in embryo samples at the same developmental stages. mRNAs and miRs were isolated in these described samples using the mirVana™ miRNA Isolation Kit according to the manufacturer’s instructions. RNA quantity and purity were determined (Nanodrop 1000, Thermo Scientific, Spain). Isolated RNA and miR were cleaned with DNase using the DNase I, RNase free kit (Thermo Scientific, Spain) for 30 min at 37 °C to remove genomic DNA contamination. For molecular analyses, only the isolated miR and mRNAs that showed high purity (A260/280 > 1.8) were employed and stored at −80 °C until qPCR analysis.

#### Reverse transcription

For miR transcription in sperm and embryo samples, a specific Taqman Small RNA probe (5x) for each miR (dre-miR-200a-5p, dre-miR-122-5p, dre-miR -141-3p and dre-miR-92a-3p) was employed following the manufacturer’s guidelines. For mRNA zebrafish embryo samples, complementary DNA (cDNA) was obtained from 1 μg RNA using the cDNA synthesis kit (Invitrogen, Spain), following the manufacturer’s protocol. The cDNA of RNA and miR samples was stored at −80 °C until further use.

#### Real-time PCR analysis

For miR analysis, reactions were carried out according to the protocol for Taqman Universal PCR master mix II (Thermo Fisher, Spain). The specific probes and amplification protocols were previously published for these transcripts in mammalian sperm^17,25^. miR-92-3p was employed as normalizer due to it being proven in previous miR expression studies in sperm^31^.The amplification protocols and primers were previously reported for these transcripts^32^. NormFinder software was employed to analyze the candidate normalization genes (beta actin, *actb2*, and elongation factor 1α, *ef1α*). In sperm analyses, miR results were expressed as the mean ± SE of the 2^−ΔΔCt^ method of three independent experiments of three different pools of males and containing 3 individual males. In zebrafish progeny analyses, 5 independent *in vitro* fertilizations were performed in each experimental group (high and low motility samples) and different developmental stages were analyzed.

#### Target prediction

The Target Scan Fish 6.2 program (http://www.targetscan.org/fish_62/) was employed^33^ for the analysis of the potential targeted mRNAS of some of the studied miRs (miR-141-3p and miR-200a-5p*)*. TargetScan predicts biological targets of miRNAs by searching for the presence of 8mer and 7mer sites that match the seed region of each miRNA. Target Scan Fish predicts targets by either the predicted efficiency of targeting (context + scores) or the probability of conserved targeting (P_CT_). Firstly, algorithm searches the sites that have full complementarity in the miRNA seed region and then they are extended to 21-23 nucleotide-long fragments representing true interactions^34^. Results are classified on the basis of length of exact matching and an occurrence of adenine at the first position of mRNA target site which seems to be evolutionally conserved and may act as a recognizing anchor for RISC^34^.

### Statistical analysis

Data were analyzed using SPSS V.22 (IBM, USA), GraphPad Prism V.5 and Microsoft Excel. For sperm motility and kinetic parameters, a general linear model with the Bonferroni correction was used (p < 0.05). Sperm quality results were expressed as mean of percentages in all analyses of 48 individual males. To analyze sperm volume and concentration, an Independent Samples t Test comparing the means of two independent groups of zebrafish males (good and bad breeders) was applied in order to determine whether there is statistical evidence that the associated population means are significantly different (p < 0.05). A Principal Component Analysis was performed to reduce the dimensionality of a data set in which there are a large number of interrelated sperm quality variables, while retaining as much as possible of the variation present in the data set. For molecular analyses, qPCR results for miR and transcript analysis in sperm and zebrafish embryos at three different developmental stages were expressed as the mean ± SE of the 2^−ΔΔCt^ method. The Student´s t-test (μ = 1) was performed according to previous studies^35^ to detect modifications in transcript levels between control group (good breeders) and studied group (bad breeders).

## Results

### Sperm quality assays

Good and bad zebrafish breeder experimental groups were established according to total motility parameter (%): good breeders presented high motility values (>70%) and bad ones recorded low motility values (<50%). Males that presented intermediate motility values were discarded from the study (Fig. 1). As expected, total sperm motility from experimental groups was significantly different between groups during the first min post activation (Fig. 2A,B). Similarly, in sperm kinetic parameters (VSL, VCL and VAP) good zebrafish breeders showed a significantly (p < 0.05) higher values throughout the first minute post activation of sperm than the bad ones (Fig. 2C). When concentration and volume were analyzed in these established groups, significant differences (p < 0.05) were found, the values obtained in good zebrafish breeders being higher than in the bad breeders (Fig. 2F,G).

When we analyzed all sperm quality parameters in a Principal Component Analysis, we found that despite of total motility, concentration and volume were the best sperm quality descriptors when we compared good and bad zebrafish breeders. Considering these results, we analyzed the correlation between these two parameters in a second analysis showing a significant correlation between them (r = 0.6441 and p < 0.0001). (Fig. 2D,E).

### Sperm molecular assays

In contrast to what was observed in the analysis of quality parameters, global methylation results using EpiJET DNA Methylation Analysis Kit (MspI/HpaII) showed no differences between experimental good and bad zebrafish breeders (Fig. 3A and Supplementary Fig. 1). A methylated status was observed in all sperm samples analyzed (Fig. 3A and Supplementary Fig. 1). When we performed the specific locus analysis to obtain information above the methylation percentage in *dmrt1* promoter, a partial methylation pattern was observed in both experimental groups (Fig. 3B). However, no significant differences were found between the studied groups (0.41% in good breeders comparing to 0.60% in bad ones) (Fig. 3B).

Concerning miR analysis using previously characterized sperm molecular markers in zebrafish and human, significant differences were found between the experimental groups. miR-200a-5p and miR-141-3p showed a significant over-expression (p < 0.05) in bad breeders comparing to good ones, being a 4-5 fold increase (Fig. 3C). Moreover, a down-regulation of miR-122-5p was observed in the bad breeder group according to previous studies in human sperm (Fig. 3C).

### Early development analyses of the progeny

**Figure 4:**
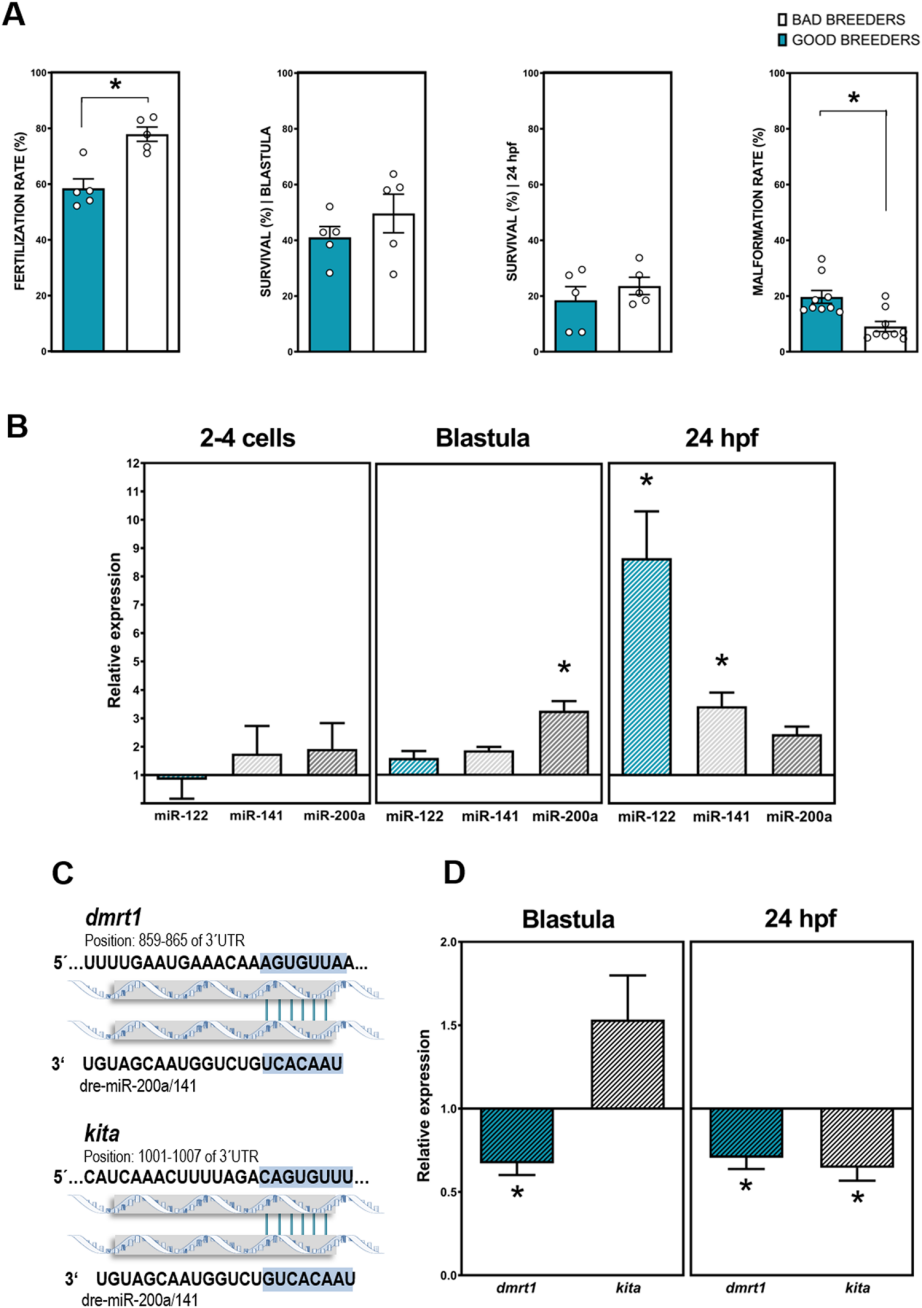
Progeny analyses from zebrafish good and bad breeders. (**A)** Percentage (%) of fertilization rate, embryo survival in blastula and 24 hpf stages and malformation rate in zebrafish progenies from good and bad breeders. (**B)** Relative expression of each microRNA calculated using the 2^−ΔΔCt^ method at three different early developmental stages (2–4 cells, blastula and 24 hpf). The figure shows expression of each microRNA in the bad breeders relative to that in good breeders, which was set to 1. Data are expressed as the mean ± SEM of 2^−ΔΔCt^ values from five independent *in vitro* fertilizations. Asterisks represent significant differences (p < 0.05) between experimental groups. (**C**) miR-200a-5p and 141-3p target analysis and the seeds matched in the 3′-UTR of each target gene. (**D**) Relative expression levels for each gene relative to the housekeeping genes actin beta 2 (*actb2*) and elongation factor 1 alpha (*ef1α*) were calculated for all samples using the 2^−ΔΔCt^ method. The figure shows expression of each gene in the progenies form bad breeders at two developmental stages (blastula and 24 hpf) relative to that progeny of good males, which was set to 1. Data are expressed as the mean ± s.e.m. of 2^−ΔΔCt^ values from three independent experiments with three replicates for each. Asterisks represent significant differences (p < 0.05) between experimental groups.

When we analyzed the fertilization rate and progeny development obtained from good and bad zebrafish breeders, significant differences were found in the fertilization success (77.84% versus 58.48% in good and bad breeders respectively) (Fig. 4A). However, non-differences were found in embryo survival at blastula and 24 hpf stages between experimental groups (Fig. 4A). In addition, the progeny of bad zebrafish breeders presented a higher percentage of embryo malformations than the progeny of good breeders (23.55% and 18.45% respectively) (Fig. 4D).

The altered miRs founded in the sperm of bad zebrafish breeders comparing to good ones were analyzed in both experimental group progenies (good and bad breeders) at 2–4 cell, blastula and 24 hpf stages by qPCR. In zebrafish blastula embryos a miR alteration was observed in blastula stage embryos from bad breeders A significant (p < 0.05) up-regulation of miR-200a-5p (3.3 fold increase) was found in zebrafish embryos from bad breeders comparing to good zebrafish breeders (Fig. 4B). In 24 hpf stage, zebrafish embryos presented significant differences (p < 0.05) between the experimental groups, being miR-122-5p and miR-141-3p upregulated (8.3 and 1.7 fold increase, respectively) in bad breeders comparing to the control ones (good breeders) (Fig. 4B).

mRNA target analysis using the TargetScanFish 6.2 program confirmed previously obtained results in zebrafish that established that miR-200 family (specifically miR-200a-5p and miR-141-3p) presents as potential targets several mRNAS involved in sperm quality, fertilization success and male sex differentiation such as: *dmrt1* and *kita* mRNAs (Fig. 4C). *dmrt1* and *kita* transcripts were analysed by qPCR comparing to good and bad zebrafish breeders in blastula and 24 hpf embryo stages. A significant (p < 0.05) down-regulation of *kita* was found in bad breeders in 24 hpf stage embryos from bad zebrafish breeders (0.64 fold increase) (Fig. 4D). In terms of *dmrt1* expression, a significant down-regulation was founded in the two studied developmental stages (Fig. 4D) of zebrafish embryos from bad breeders (0.67 and 0.7 in blastula and 24 hpf stages respectively).

## Discussion

The decrease in human sperm quality during recent years is quite controversial, several articles stated this is a fact^36–38^ but others claim those results should be analyzed with caution^39,40^. However, a good indicator of human sperm quality decrease tendency is the fact that World Health Organization (WHO) has systematically downgraded the previous values (volume, sperm concentration, total sperm number, morphology, vitality and progressive motility) to consider a sperm sample noormozoospernmic^41^. Some studies point to a decrease in sperm quality due to a combination of several lifestyle factors^41^. It is well known that obesity has become a problem in some regions of the world and it has been demonstrated that it also has negative effects on sperm quality^42,43^. Moreover, negative effects on sperm quality due to increased paternal age has been demonstrated, which is a frequent phenomenon in recent year’s^16,44^. Poor sperm quality can clearly have negative effects on fertilization and embryonic development but it is also crucial to know if it could leave a permanent mark on progeny in a similar way that paternal toxic exposure does^45–47^. In order to explore this, we have used zebrafish as model species. This small teleost has been considered an optimal model for reproductive studies with potential extrapolated results to mammal species, including humans^21,48,49^.

The first challenge of this study was to properly define the experimental groups: good and bad breeders. Different sperm parameters should be considered in the analysis of a specific sperm sample and these parameters have been widely discussed in fish by several authors^30,50,51^. In the case of humans, some factors such as sperm motility, concentration and morphology should be taken into account together to define the fertilization success^52,53^. However, total sperm motility has been considered the most important parameter in fish sperm quality determination due to its high correlation with fertilization success in different species^28,30^. Having this in mind, we classifed bad and good breeder attending to total motility (Fig. 1). We found significant differences in all parameters analyzed: kinetic parameters (Fig. 2C), volume (Fig. 2F) and concentration (Fig. 2D,G) between experimental groups. In addition, the concentration parameter was determined as the most principal component of the obtained variance in sperm samples between the experimental groups (good and bad zebrafish breeders) (Fig. 2H) and as expected a high correlation between total motility and concentration was detected (r = 0.6441 and p < 0.0001) (Fig. 2E) showing up these parameters as the best quality markers to establish our experimental groups. In terms of sperm molecular analyses, we analyzed global methylation pattern in the sperm samples of the studied groups and non-significant differences were found (Fig. 3A). A complete methylation status was observed in all sperm samples analyzed, according to what could be expected in a normal transcriptionally inactive sperm cell with high condensed DNA^54,55^. In terms of specific promoter methylation analysis, *dmrt1*, a sperm quality marker in fish^32^, was analyzed. This selection was done considering our previous studies in fish, where *dmrt1* was selected as a good molecular marker of sperm quality in good breeders^32^. Moreover, this transcript has been described as necessary for male sexual development in human and zebrafish^56,57^. Non-significant differences were found between experimental groups presenting a partial methylation pattern in this promoter (Fig. 3B). According to these results, we found no evidence of alteration of global methylation status in poor quality sperm samples and for this reason methylation was not further evaluated in the embryos. These results do not exclude the possibility of having potential differences at other levels such as methylation differences in particular promoters.

miR analysis in sperm samples, reported significant differences in all studied miRs between zebrafish experimental groups (Fig. 3C). These results are in accordance with those obtained in human^25^. The studied miRs were previously characterized as potential sperm quality molecular markers in humans because their dysregulation was associated to oligoasthenozoospermia or asthenozoospermia. In particular, miR-200a-5p and miR-141-3p were up-regulated in the case of oligoasthenozoospermic and asthenozoospermic patients and miR-122-5p was down-regulated in these patients^17,25^. miR-122 is enriched in germ-cells at later developmental stages^58^. In this study, authors provided evidences that miR-122 changes are attributable to early events in spermatogenesis^59^. These findings suggested that cellular miRNA content of mature germ-cells depends heavily on the efficacy of the spermatogenic process, which is crucial for the determination of miRNA molecular signature of spermatozoa^59^. Moreover, more recently, miR-200 family, was defined as sperm motility marker in zebrafish^26^ and both miRNA, miR-200a-5p and miR-141-3p belong to this family. Once the molecular differences between good and bad breeders in terms of miRs were corroborated, *in vitro* fertilizations were performed. As expected, we observed significant differences in fertilization success using bad and good sperm quality samples (Fig. 4A). However no significant differences in survival were found in blastula and 24 hpf embryos between groups (Fig. 4A). These results are in accordance with previous findings in other fish species where embryos with initial abnormal cleavage had, in later developmental stages, similar hatching rates than control embryos^60^. However an increase in embryo malformations was recorded in the progenies of bad breeders, as confirmed in the progeny phenotypic analysis (Fig. 4A).

In order to explore whether the altered sperm miRNA signature is conserved in the developing embryo we analyzed the same set of miR altered in poor sperm quality samples in the embryos.

We did not detect any significant differences among experimental groups in miRNAs content in 2–4 cell stage embryos (Fig. 4B). In this stage, the level of relative expression of the studied miRs was lower comparing to the other developmental stages. The low presence of miRNAs in this stage has been also reported in other zebrafish studies^61^. However, we found a significant alteration in some miRNAs in blastula and 24 hpf: miR-200a-5p (in blastula) and miR-122-5p and miR-141-3p (in 24 hpf) were differentially present in those embryos fertilized with good quality sperm samples and those fertilized with bad quality samples (Fig. 4B). The function of miR-200a in zebrafish early development has been previously studied. Over-expression of miR-200s (miR-200a and 141) in early stages of development triggers cell cycle arrest and induced apoptosis, thereby inhibiting somatic growth of zebrafish embryos^61^. In this work, authors demonstrated that these miR-200s regulate body size regulating cell proliferation and apoptosis by a negative interaction to growth hormone/ insuline-like growth factor axis^61^. The expression of miR-122-5p in the 24 h embryos was significantly increased in the progenies derived from bad male breeders. These results contrasted with the lower presence of this miRNA in bad quality samples, both in zebrafish (Fig. 3C) and human sperm^17^. Despite the discrepancy in the amount of this particular miRNA between sperm and the resulting embryo, this altered pattern clearly indicate that fertilization with low quality spermatozoa could produce molecular alterations in progeny. However, we decided to continue our study analyzing only the targets of those miR that present the same tendency in the expression pattern in spermatozoa and embryos.

In order to corroborate whether the targets of the altered miR were also affected, a target analysis of these altered miRs was performed. For expression studies, we selected the targets of miR-141-3p and 200a-5p. These miRNAs belong to the same family and among their targets two genes defined as molecular markers of good and bad breeders in our previous studies^32^ were found: *kita* and *dmrt1*. Moreover, these molecular markers are involved in male sexual development in zebrafish^56^, sperm quality and early embryo development^32^. *dmrt1* have been reported to be associated with spermatogenesis and sperm capacity in zebrafish^26^ and an aberrant expression of these factors results in impairment of sperm activity and male infertility^26^. Interestingly, in a recent study published in this model, then authors examined in detail the role of *dmrt1* in sex determination and gonad development^56^. In this study, *dmrt1* mutants developed as females were fertile demonstrating a successful male-to-female sex reversal, however, a mutants developed as males (a lower percentage) were sterile and presented testicular dysfunctions. Therefore *dmrt1* has a critical role in male sex determination and testis development^56^. A down-regulation of these two transcripts was found in both developmental stages embryos resulting from fertilization with bad quality samples (Fig. 4D). The differences between experimental groups were more consistent in 24 hpf stage (2 altered transcripts) than in blastula stage (1 altered transcript).

This study allow us to conclude that fertilizing with low sperm quality samples has direct consequences on fertilization rates, but also produces higher malformation rates and leaves molecular marks in those embryos that survive. The importance of fertilizing with high sperm quality samples becomes relevant from a new perspective: to avoid molecular alterations in the progeny that could remain masked and could have different unexpected consequences in it.

## Supplementary information

Supp Fig 1
