# Supplementary material for: Effect of low sperm quality on progeny: a study on zebrafish as model species
Source: Sci Rep. 2019 Aug 1;9:11192. doi: 10.1038/s41598-019-47702-7 (PMC6671952; doi:10.1038/s41598-019-47702-7)

Effect of low sperm quality on progeny: a study on zebrafish as model species.

Marta F. Riesco, David G. Valcarce, Juan Manuel Martínez-Vázquez, Vanesa Robles

SUPPLEMENTARY FIGURE 1

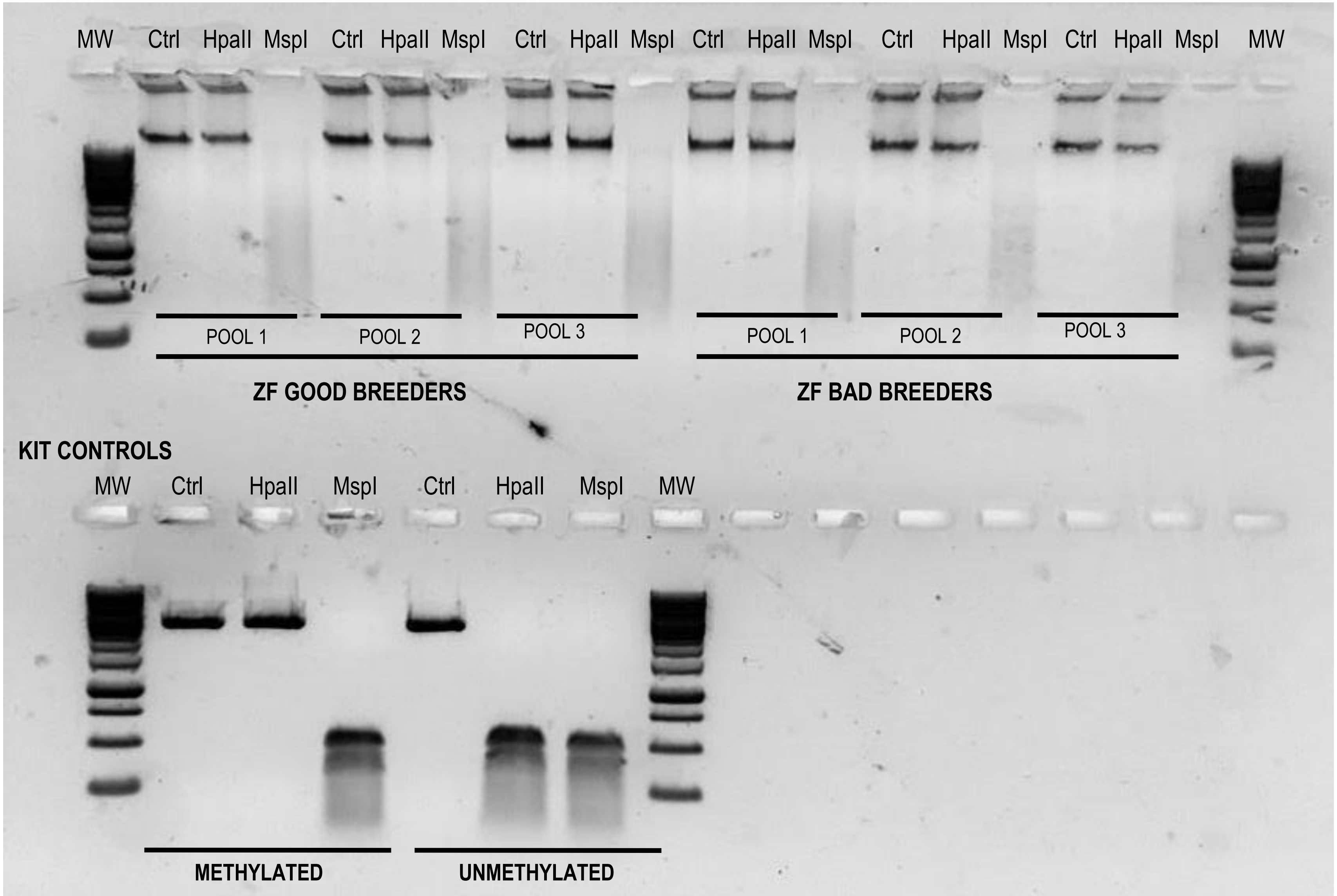

Supplement: Supplementary file 1 — Supp Fig 1 [file 41598_2019_47702_MOESM1_ESM.pdf]
